# Malaria in Europe: A Historical Perspective

**DOI:** 10.3389/fmed.2021.691095

**Published:** 2021-06-30

**Authors:** Mahmoud A. Boualam, Bruno Pradines, Michel Drancourt, Rémi Barbieri

**Affiliations:** ^1^IHU Méditerranée Infection, Marseille, France; ^2^Aix-Marseille University, IRD, MEPHI, IHU Méditerranée Infection, Marseille, France; ^3^Unité parasitologie et entomologie, Département microbiologie et maladies infectieuses, Institut de recherche biomédicale des armées, Marseille, France; ^4^Aix-Marseille University, IRD, SSA, AP-HM, VITROME, Marseille, France; ^5^Centre national de référence du paludisme, Marseille, France; ^6^Aix-Marseille University, CNRS, EFS, ADES, Marseille, France

**Keywords:** Malaria, *Plasmodium*, intermittent fever, European, paleopathology, paleomicrobiology, quinquina, quinine

## Abstract

Endemic malaria, which claimed 229 million new cases and 409,000 deaths in 2019 mainly in Africa, was eradicated from Europe by the mid-20th century. Historical descriptions of intermittent tertian and quartan fever reported in texts of Hippocrates in Greece and Celsus in Italy suggest malaria. A few paleomicrobiology investigations have confirmed the presence of malarial parasite *Plasmodium falciparum* in 1st, 2nd, and 5th century infected individuals in diverse regions of Italy, and *Plasmodium* sp. later in Bavaria. The causative *Plasmodium* pathogens, discovered in the 19th century in Algeria, were controversially used as therapeutic agents in the European pharmacopeia more than two centuries after effective quinine-based treatments had been introduced in Europe. How Europe managed to eradicate malaria and what the history of malaria was in Europe are of medical interest, and this review traces research pathways for a renewed understanding of malaria eradication in Europe through combined historical and paleomicrobiological investigations.

## Introduction

Malaria is a vector-borne disease in which *Plasmodium* spp. causative pathogens are transmitted via the bite of the infected female *Anopheles* mosquito (1). Malaria remains the single most prevalent life-threatening infectious disease in the world according to the World Health Organization (WHO) (2). The genus *Plasmodium* is composed of more than 250 species, but only five species *Plasmodium falciparum, Plasmodium vivax, Plasmodium ovale Wallikeri, Plasmodium ovale curtisi*, and *Plasmodium malariae* (3) are shown to be involve in human-to-human transmission after *Anopheles* bites (4). Three simian parasites *Plasmodium cynomolgi, Plasmodium inui*, and particularly *Plasmodium knowlesi* (5) are known to be responsible for human malaria (4).This last species is the most common cause of human malaria in Malaysia. Malaria reservoirs have been eradicated in Europe since the 20th century, which currently experiences only imported cases. However, according to historical texts and medical, paleomicrobiology and paleogenetic data, malaria may have played a key role in the history of past Europeans.

The oldest evidence for the *Plasmodium* parasite (i.e., *dominicana* n. sp.) was found in amber dating back 30 million years in the Dominican Republic (6). Most probably malaria then co-evolved with non-human primates in Africa: *P. falciparum* may have emerged from gorilla parasites about 10,000 years ago, while *P. vivax* may have emerged much earlier from non-specific Apes hosts (4, 7–9). On the other hand, *P. knowlesi* probably arose in Southeast Asia among macaque monkeys about 478,000–98,000 years ago (10), while the origins of *P. malariae* and *P. ovale* remain uncertain, although these parasites are currently associated with gorilla, chimpanzee, and bonobos in Africa (4). Malaria co-evolved over millennia alongside humans and reached tropical and temperate climatic areas of the Old World (9, 11). Malaria may have moved westward from India to Europe along with prehistoric populations (12). During the Upper Paleolithic, malaria may not have had a significant selection force, in agreement with its putative recent introduction in European populations (12). Another hypothesis regarding the introduction of malaria in Italy, relies on the introduction of *Plasmodium* vectors from North Africa (13) in the frame of Sardinia invasion by Carthaginians, 7th to 2nd century BC (13, 14).

As for the term malaria, it derives from the Italian word *mal'aria*, meaning “bad air” (15). In the Middle Ages, malaria was thought to be transmitted by humid and stale air, especially in swamps and marshes. Interestingly, the Greek word for malaria (elonosia) literally means “the disease of the marsh” (16). The term malaria was introduced in England by Horace Walpole in 1740, following his letters from Italy, but it was not until 1827 that the term “malaria” was first used in an English scientific publication (17).

### Discovery of *Plasmodium* Pathogens

**Figure 1:**
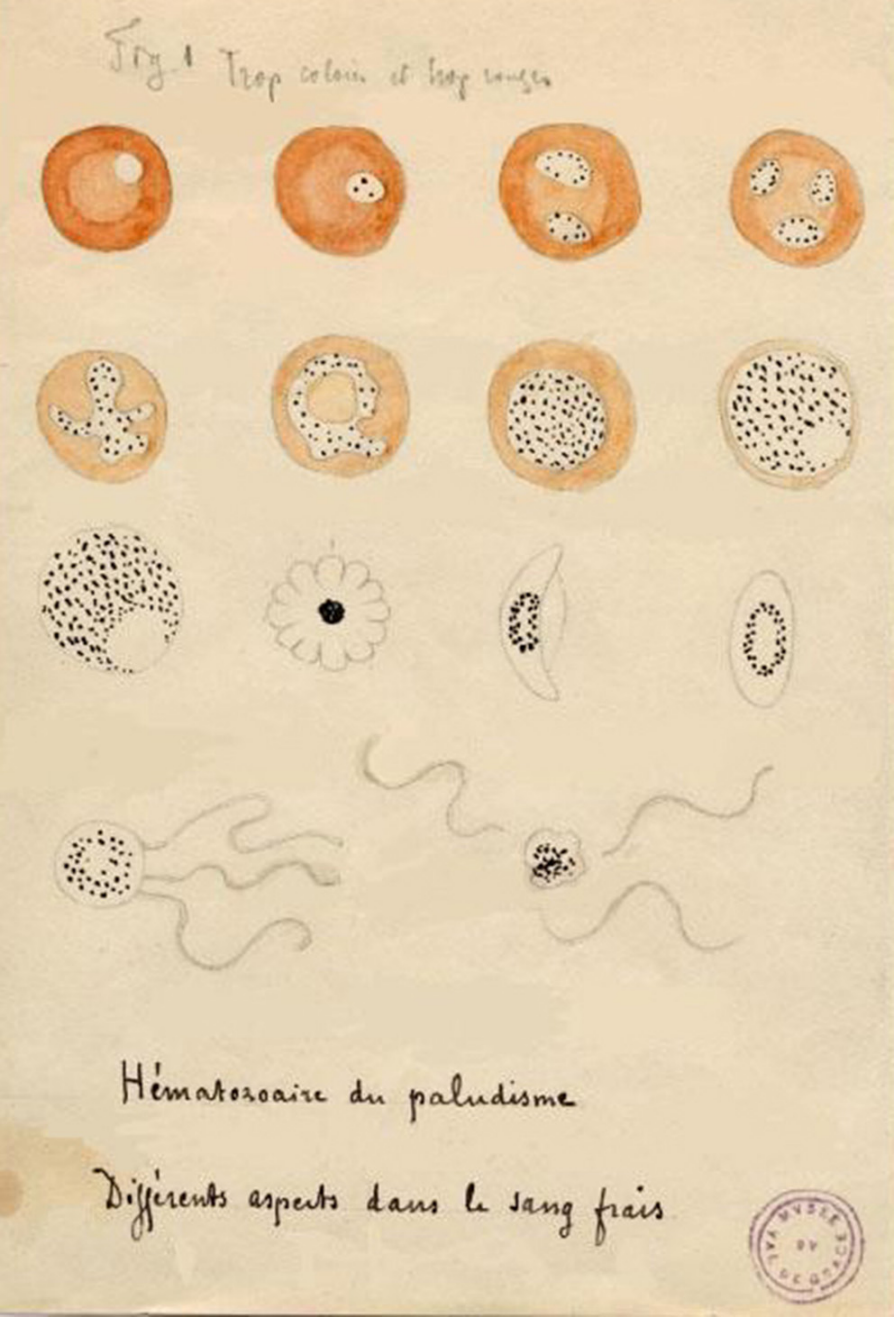
Drawing by Alphonse Laveran of the different aspects of hematozoan malaria' (21).

The first microbiological studies of malaria began at the end of the 19th century, during the birth of microbiology. The interest of European scientists in malaria was reinforced by the development of large colonial empires and the need to protect colonists and populations from this deadly infection (11). A French army surgeon, Alphonse Laveran, first observed *Plasmodium* gametocytes in patients' blood by optical microscopy in 1878 in Bône, Algeria (18). On November 6th, 1880, A. Laveran observed gametocyte exflagellation while working at the hospital in Constantine, Algeria; he noted the presence of *Plasmodium* parasites in the fresh blood of a 24-year-old soldier who had been febrile since October 10th, 1880 (19, 20) and this observation is regarded as the first one of *P. vivax* (18) (Figure 1). Laveran demonstrated that “l'impaludisme,” as it was called at the time, was not of bacterial but of parasitic origin (18). He wrote: “From then on I no longer had any doubts about the parasitic nature of the elements that I had found in palustrine blood” (18). Further discovery of the mosquito as the exclusive vector of malaria followed from the intuition of Patrick Manson, who had previously demonstrated that other blood parasites, such as certain filarial worms, were transmitted by mosquitoes (22). From this initial discovery, Manson assumed that mosquitoes could also be the vector for malaria parasites, thus associating the relationship between disease and swampy areas overpopulated by mosquitoes (22). In 1897 in India, British army surgeon Ronald Ross discovered that *Plasmodium relictum* was transmitted by culicine mosquitoes (20), and demonstrated 2 years later that human malaria parasites could be transmitted by *Anopheles* mosquitoes. In fact, in 1898 Italian malariologists Giovanni Battista Grassi, Amico Bignami and Giuseppe Bastianelli had already provided the formal proof that malaria parasites could be transmitted from man to man via *Anopheles* mosquitoes (23, 24). Within 2 years following this discovery, they further understood that only *Anopheles* females could transmit malaria parasites and described the complete cycle of *P. vivax, P. falciparum* and *P. malariae* in the mosquito (20). In 1947, Henry Shortt and Cyril Garnham demonstrated pre-erythrocytic schizonts of *Plasmodium cynomolgi* in the liver of a Rhesus monkey, and finally in 1982, Krotosky deciphered the dormant exo-erythrocytic hypnozoites of *P. vivax*, explaining malaria relapses (20). It thus took more than one century of work carried out by hundreds of scientists to elucidate and fully understand the complete cycle of human malaria parasites (24, 25).

### History of Fever in Europe

#### Antiquity (Before 476 AD)

According to historical sources, malaria may have been described for the first time during the fourth and fifth centuries BC in ancient texts, such as the Hippocratic Corpus, which mentioned a disease characterized by intermittent fevers (26). Hippocrates (460-370 BC) described episodes of benign tertian fever that have been interpreted as *P. vivax* malaria, while the term quartan fever could refer to *P*. *malariae* malaria (27). The Greek physician used the term “dangerous fumes” emanating from the ground that were carried by the wind to describe the mist which he believed caused serious illness (26). Hippocrates then linked the occurrence of climatic phenomena and the variation of environmental conditions to the occurrence of episodes of intermittent fevers and classified fevers according to their periodicity: tertian fever from the Greek tritaios pyretos (intense fever every 3 days) and quartan fever from Greek tetartaios pyretos (intense fever every 4 days) (26). He reported the seasonality of the disease, the proximity of cases to standing water and the use of antimalarial agents (28). These semi-tertian fevers, like other intermittent fevers, were common during the fifth century BC in certain localities of northern Greece such as Macedonia, and in Laconia (13, 16), before spreading throughout Western Europe. Apart from historical sources, there were very few reliable data concerning the possible presence of malaria during this period. However, malaria parasites may have exerted an evolutionary selection shaping the human genome, including genes for immunity, cell adhesion, and inflammation (29). However, genomic analysis of 224 human European genomes dating from the Upper Paleolithic to Roman periods have not detected specific malaria resistance mutations in genomes, such as mutations affecting the *G6PD* gene (glucose-6-phosphate dehydrogenase), *HBB* gene (hemoglobin subunit beta), or Duffy blood group, suggesting a weak adaptation to malaria or a mild form caused by *P. vivax* in Europe during the Prehistory, Protohistory, and Antiquity periods (12). During the Roman period, many outbreaks of deadly fever were reported, the most significant of which took place in the 1st−2nd century AD and which may have been caused by *P. falciparum* circulating in Italy at that time (30). Spleen remedies created by the physician Pedanius Dioscorides may have been developed in response to the endemic malaria plaguing Roman populations (31).

#### Middle Ages (476- 1492 AD)

Climatic and environmental variations with successive periods of warming and cooling may have influenced the emergence of certain infectious diseases on the European continent (32). Growing urbanization in Eurasia at the beginning of the Middle Ages favored pandemics through increased density of human contacts (32). The medieval population undoubtedly experienced malaria epidemics, not only in the Mediterranean region but also in Northern France, Germany and England. The most interesting historical sources are from England and Italy, while French sources suggest that certain regions were endemic for malaria in France and Italy between the 6th and 9th centuries (33). Old texts citing intermittent fevers, whether tertian or quartan, are interesting clues in tracing the history of malaria in medieval Europe. According to descriptions in these texts, researchers refer to the notion of benign tertian fever as an indicator of infection with *P. vivax*, and quartan fever of *P. malariae*, while the term malignant tertian fever may have referred to *P. falciparum* malaria (13, 34). However, the unique European species (Italy) of malaria identified by paleomicrobiology belonged to *P. falciparum* during Antiquity. In addition, the reservoirs of malaria in Europe are poorly identified and differ depending on the period and climatic variations.

Malaria probably took root in certain niches around marshes, as in Italy, creating an environment conducive to the spread and development of *P. falciparum* between the end of Antiquity and the beginning of the Middle Ages (35).

#### Modern Era (1492- 19th Century)

Due to the accumulation of several factors, such as climatic variations and the occurrence of several wars during the 17th and 18th centuries, the incidence of malaria believed to be caused by *P. vivax* and *P*. *malariae* was high (36). In France, the use of quinquina powder since the 17th century to treat intermittent fever may trace malaria epidemics (Box 1–**3**). A study carried out by the Royal Society of Medicine counted 820 cases of tertian fever, 230 cases of daily fever and 127 cases of quartan fever in the marshy areas of the Paris region from 1783 to 1785 (37).

Box 1 **Quinine, a historical treatment of malaria.**

**Figure 2:**
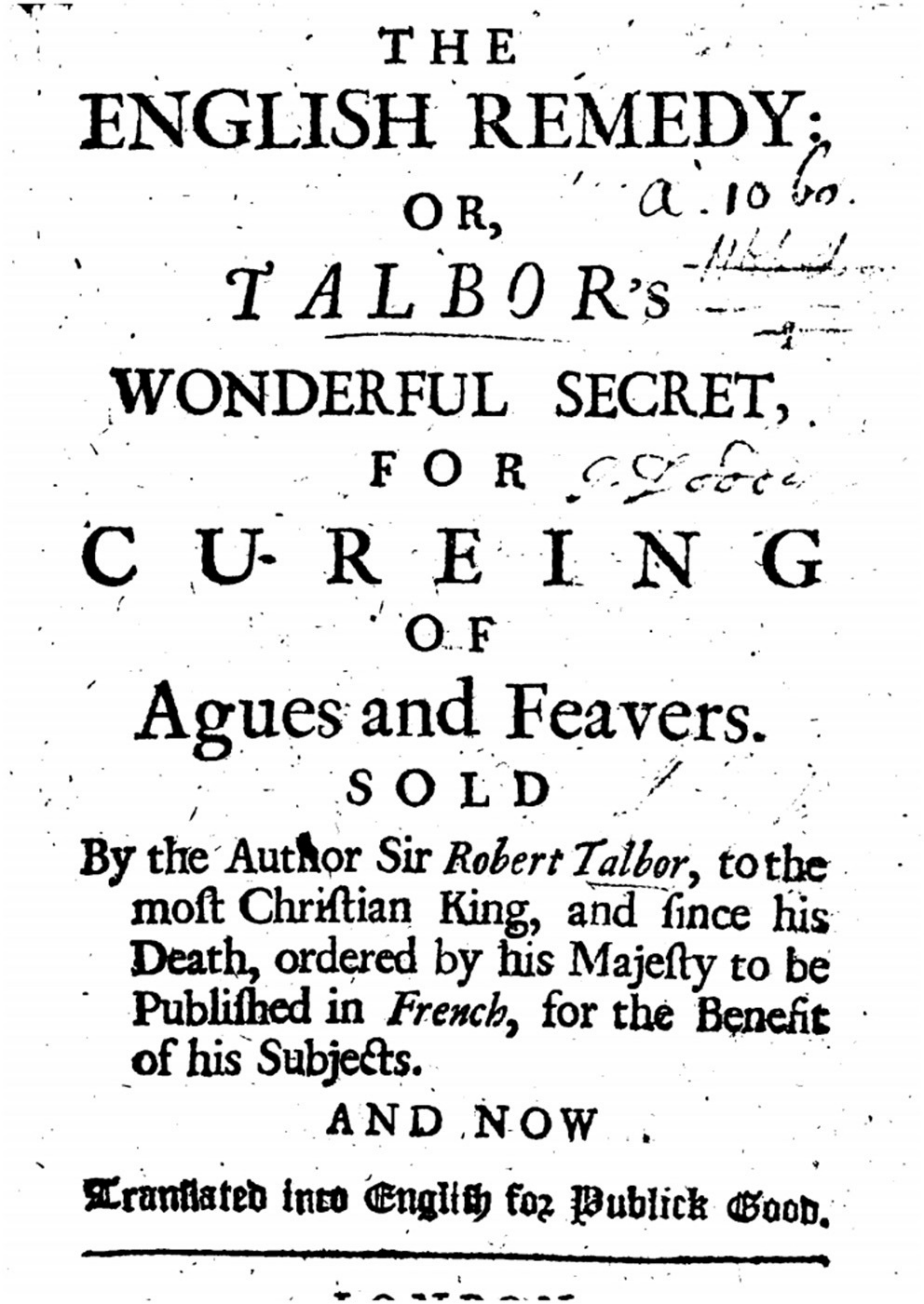
The english remedy: Talbor's wonderful secret for curing of agues and feavers (47).

**Figure 3:**
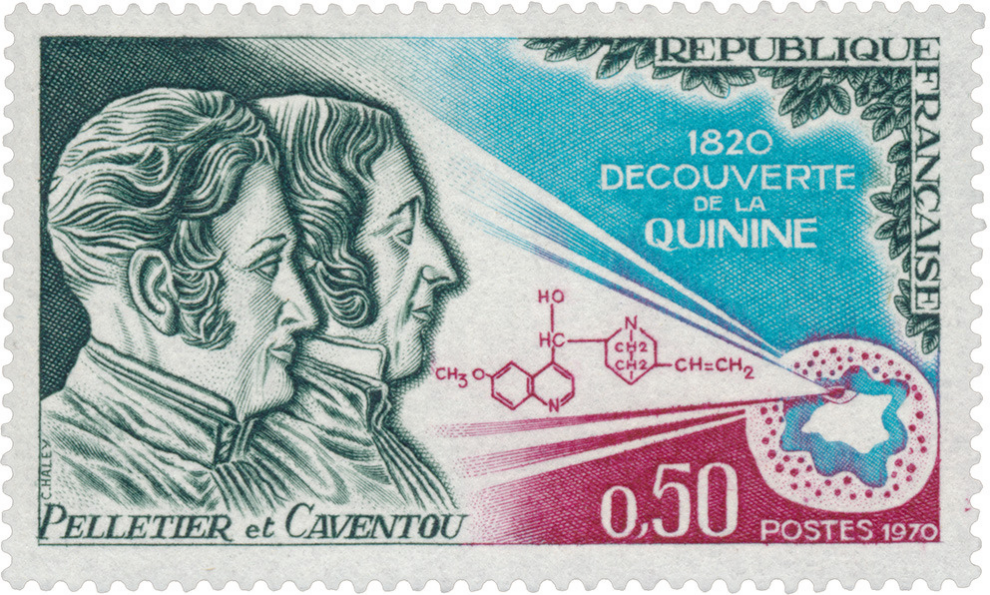
Postage stamp edited in 1970 with the images of Pelletier and Caventou, who first isolated the active ingredient “quinine”.

Quinine, a natural compound extracted from the bark of *Cinchona* trees, was used historically in the high plains of South America. The genus *Cinchona* now includes 21 different species and the precise composition of quinine varies, depending on the tree species from which it has been extracted (44). Quinquina powder was cited by Europeans under different names, including Peruvian bark, Jesuit powder, *Cinchona*, quinquina, kinquina, kinkina, and the English remedies. The use of quinquina as an antipyretic drug was reported in the early 17th century by Father Antonio de la Calancha in a book he published in 1638 in Barcelona (44). Use of quinquina powder then declined, as it was believed to be ineffective or dangerous because of the confusion between real *Cinchona* bark and different barks of different species of the genus *Cinchona* (some species are devoid of quinine and others have very little quinine) (45). The apogee of quinquina powder followed its use by the English pseudo-apothecary Robert Talbot (also known as Talbor) who gained notoriety after he cured Charles II, King of England (46, 47) (Figure 2). The active ingredient of the quinquina bark, named quinine, was first isolated in Paris in 1820 by two French pharmacists, Pierre-Joseph Pelletier (1788–1842) and Joseph Bienaimé Caventou (1795–1877) from the bark of *Cinchona succirubra* (red Cinchona) (48) (Figure 3). Quinine was heavily used during World War I (1914–1918) by British, French and German physicians, both as a prophylaxis and as a treatment for malaria (49).

Box 2 **Malarial fevers at the court of french king louis XIV.**

More than 36,000 people worked in the construction of the royal palace in the swampy site of Versailles, and some contracted intermittent fevers. According to historical sources, several thousand workers may have died of malaria working on the construction of the basins in the park of Versailles (50).

The Duke Saint-Simon evoked in his memoirs the marshes surrounding the castle of Versailles during the construction and speaks of an unhealthy humidity (51). Under the reign of Louis XIV, swamps were already considered as sources of disease, and the term “miasmas” began to be used to describe “bad air” carrying disease (51). In Versailles, several inhabitants of the palace experienced intermittent fevers, notably in 1680 when the eldest son of the Sun King, Le Grand Dauphin and his daughter La Dauphine had attacks. The King summoned Robert Talbot, who thanks to the administration his remedy based on *Cinchona* powder, cured Le Grand Dauphin in 4 days. Very impressed by this cure, the king paid him 48,000 pounds, in addition to an annual annuity of 2,000 pounds, for the secret recipe for Talbot's remedy (51). The close relatives of Louis XIV used to drink quinquina too, and Saint-Simon refers in his memoirs to the taking of quinquina in Versailles by M. de Beauvilliers to stop a stubborn fever before going to join the new king of Spain, Philip V (52). Saint-Simon was cautious using quinquina (53), and advised his host M. de Chevreuse, who was taking a glass of quinquina to relieve gastric pains, on the risk of perforating his stomach if he took quinquina without eating (52).

During the 16th to 19th centuries in southern and eastern England, known to be very marshy areas, a high mortality rate occurred in the population, which according to old texts was due to fevers, termed “marsh fever” (38). One study suggested that swamp fever was actually malaria, assuming that the malaria species during this time were *P. vivax* and *P. malariae* (38). These fevers were often reported in temperate regions in Europe during the summer and autumn, agreeing with the hypothesis of malaria (39). The English word for malaria in the Middle Ages was “agues” and this term was continuously used until the causative *Plasmodium* sp. was discovered (39). William Shakespeare (1564–1616), mentioned fever in several of his plays and in the poem of Venus and Adonis: “As burning fevers, agues pale and faint, life-poisoning pestilence and frenzies wood, the marrow-eating sickness, whose attaint disorder breeds by heating of the blood; Surfeits, imposthumes, grief, and damn'd despair, swear nature's death for framing thee so fair,” as a testimony of the possible impact of malaria in England. In the 19th century malaria reached Northern Europe, affecting Finland, most probably from foreign workers during construction of the Saimaa canal and railway (40). *P. vivax* malaria became common in South Finland and Scandinavia until the 20th century (40). The Italian island of Sardinia had a reputation to be unhealthy because of malaria, recording a death rate six times higher than in the rest of Italy with around 300 deaths per 100,000 inhabitants during the period from 1887 to 1902 (41).

#### Malaria and World War I

In addition to bullets and gas, soldiers from both sides involved in World War I had to deal with invisible enemies such as infectious diseases. In addition to *Bartonella quintana*, malaria affected more than 1.5 million for a fatality case of 0.2–0.5%. Problems inherent to this conflict, such as such as long periods of immobility in trenches, troop movements, and soldiers' activities have amplified the numbers of epidemics in endemic malaria zone such as Italy, Macedonia, Mesopotamia, Palestine, or England (42). In 1917, 70,000 cases of malaria were reported in the British forces alone (43). After the war, malaria spreader-emerged among the civil population in malaria free areas like northern Germany, eastern England and Italy due to the return of soldiers to their homeland and to refugee movements (42, 43).

### Paleomicrobiological Confirmation of Historical Malaria

Paleomicrobiology is a demonstrative field of research and practice, allowing the analysis of microorganisms responsible for past epidemics (54). Ancient texts provide starting points for the investigation of past infectious diseases, putting paleomicrobiological researches in an appropriate historical, ecological and social context (55). Paleomicrobiology was born in 1993 thanks to the work of Spigelman and Lemma with the detection by molecular biology of *Mycobacterium tuberculosis* DNA from an ancient human skeleton (56). The objectives of paleomicrobiology are, of course, the molecular diagnosis of ancient infectious diseases, but also tracing the genetic evolution of microorganisms, the temporal, and geographical reconstitution of ancient microbes (57).

Ancient DNA (aDNA) may persist until 1.6 Millions of years (58) with an estimated half-life of 521 years (59, 60); depending on environmental conditions which can reduce or inhibit the degradation of aDNA such as freezing or rapid postmortem desiccation. Chemical modifications that degrade aDNA are depurination (cleavage of glycosylated N bonds eliminating the purine bases) which results in aDNA fragmentation in <100-bp fragments (61–63) and an overrepresentation of purine bases at both ends of the aDNA strands (64); and the deamination of cytosines, which results in the loss of an amino group by the cytosines then converted into uracil and sequenced as thymines (61). These specific degradations make it possible to distinguish aDNA from modern DNA and its possible contamination (65, 66).

#### Paleo-Immunological Diagnosis

An initial paleomicrobiological study detected the *P. falciparum* histidine-rich protein-2 antigen (PfHRP-2) in seven Egyptian mummies from different periods, ranging from 3200 BC to 350–500 AD (67). Likewise, by using PfHRP-2 assay, *P. falciparum*. was detected in 30/71 (42%) of mummies from 3200 BC preserved in the Anthropological and Ethnographic Museum of Turin, with the same magnitude of malaria prevalence in endemic regions (68). An immunochromatographic and immunohistochemical study performed in 2008 by Bianucci et al. on muscle and skin samples collected from an Egyptian mummified child aged 15–18 months yielded positive detection of *P*. *falciparum* histidine-rich protein-2. Moreover, the muscle sample yielded positive results for the MSP1-19 antigen common to the genus *Plasmodium* (69). In Italy, studies based on immunological tests on bone samples from the Medici family of Florence, dating from the 16th century, detected proteins of *P. falciparum* in four members of the family (70). Also, an immunological study targeting the *P. falciparum* highly specific HRP-II protein on 34 individuals dating from 14th BC to the 16th century, in Sardinia indicated that malaria may date back to the Carthaginian period (502 B-C) (14).

#### Biomolecular Diagnosis

**Figure 4:**
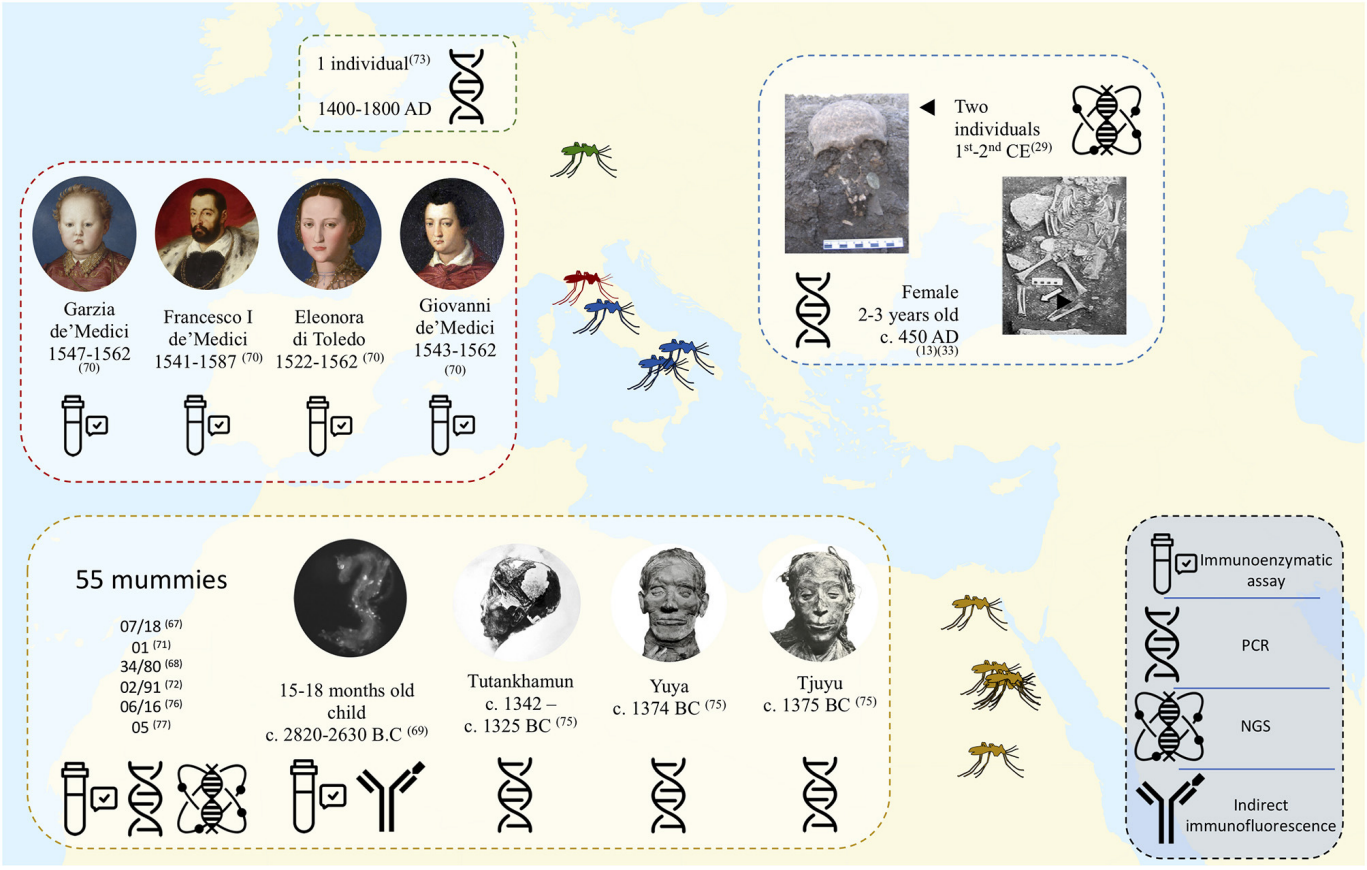
Map showing the major works carried out for the diagnosis of ancient malaria. all identified *Plasmodium* belong to the species *falciparum* except for one individual tested in Bavaria (73) and one Egyptian mummy (71) whose pathogen is *Plasmodium* sp.

**Figure 5:**
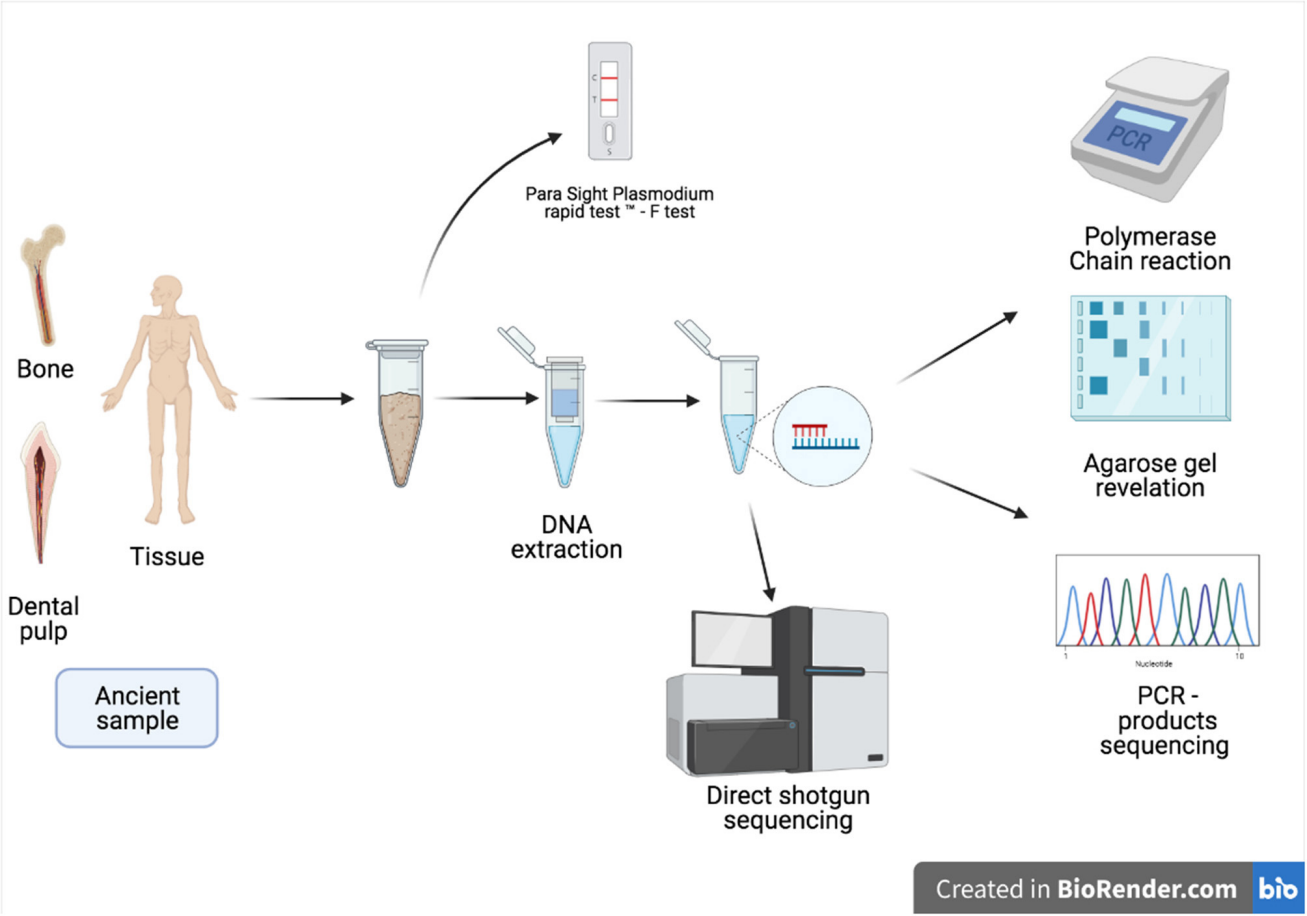
Schematic presentation of the techniques used in the diagnosis of malaria in ancient samples.

In 1997, the work by Taylor et al. pioneered the field of ancient malaria, with the development of a hemi-nested PCR diagnostic method targeting the plasmodial 18S rRNA gene in tissues of a Granville mummy dating from 700 BC. This PCR assay performed on a mummy positive for *P. falciparum* by PfHRP-2-based immunological test yielded negative results, as did one of the two positive controls introduced in the experiment (71). The technique developed by Taylor et al. was later taken up by other researchers, notably in 2001 by Sallares and Gomzi in Italy (35). These two researchers investigated an infant cemetery in Lugnano, Italy dating from 450 AD, where archaeologists suspected that malaria was responsible for the deaths. Investigating bone samples, PCR yielded positive results for *P*. *falciparum* in two samples belonging to the same individual (35). According to these data, the authors suggested the occurrence of a massive *P*. *falciparum* infection, since a positive result requires a very high level of parasitemia. Another European study occurred in 2001 by Zink et al. in southern Germany on the bones of 20 individuals dating from 1400 to 1800 AD, where the presence of an 18S rDNA sequence of *Plasmodium* sp. was confirmed in one individual (72, 73). Shotgun Illumina sequencing of DNA extracted from microscopic slides prepared between 1942 and 1944 by a Catalan center from the Ebro Delta, Spain, yielded a *P. vivax* genome exhibiting >89% alignment with the reference strain, and demonstrated variations linked to antimalarial drug resistance in this European strain (74). Studies performed on mummies of King Tutankhamun's family identified *P. falciparum* malaria as the probable cause of death of Tutankhamun and his relatives Yuya and Tiyi (Figure 4). The results were positive for malaria by PCR methods specific to *P*. *falciparum* targeting the *STEVOR, AMA1*, and *MSP1* genes and negative for other tested infectious agents (plague, tuberculosis, leprosy and leishmaniasis). These results suggest that the most probable cause of death for Tutankhamen was an avascular bone necrosis associated with *P*. *falciparum* malaria (75). In 2013, PCR-sequencing investigation of soft tissue biopsies collected from Egyptian mummies dated from 806 BC to 124 AD yielded 6/16 positive for the *P. falciparum* AMA1 gene, and four of these mummies also proved positive for PCR-based detection of tuberculosis (76). In addition, next generation sequencing (NGS) technology in 5 random mummies confirmed *P. falciparum* DNA sequences (77). In 2008, Nerlich et al. used hemi-nested PCRs targeting malaria 18S rDNA to detect *P. falciparum* in tissue samples from Egyptian mummies of different pharaonic dynasties (ranging from 3500 to 500 BC); the authenticity of PCR products was assessed by sequencing (72). This study yielded a lower incidence of malaria in ancient Egyptian mummies than that previously recorded using paleo-immunological techniques (Figure 5) (72). In fact, these contradictory results questioned the usefulness of these methodologies (67). In particular, this method, based on the detection of monoclonal antibodies to the soluble species-specific antigen PfHRP-2 (ParaSight-F) in blood, exhibited a high error rate after detection by positive PCR tests; 60% false positives were detected (78). False positive reactions using this test can be explained by cross reaction with rheumatoid factor (RF) in the blood (79).

### Paleopathology

Besides molecular diagnostic methods, direct diagnostic evidence suggests a causal link to malaria with skeletal lesions due to severe anemia resulting in a typical hyperostosis of the orbit (cribra orbitalia). Some research in paleopathology supports the hypothesis that cribra orbitalia is associated with anemia caused by infection with parasites of the genus *Plasmodium*, which other researchers therefore consider as a sign of malaria (80). In a study by Massa et al. in 2000 on Egyptian mummies, skeletal pathology was observed suggesting severe anemia in mummies testing positive for *P*. *falciparum* by immunological test. Anthropologists have indicated that the frequency of orbital cribra is very high (92%) in subjects positive for *P*. *falciparum*, suggesting that malaria was probably an important cause of anemia (68). In 2014, a study investigating 4,760 individuals from 29 ancient sites in the Nile Valley in Egypt, the prevalence of orbital cribra was 42.8%, with a non-correlation of proportion between age groups and sex, suggesting an infectious factor causing hemolytic anemia. These results suggest that malaria infection is responsible for cribra orbitalia, which can be proposed as an indication of possible malaria infection in ancient samples (81). Concerning Europe, skeletal investigation conducted in a Greek population dating from the 5th to 3rd centuries BC in the vicinity of the Black Sea hypothesized that malaria was endemic, which would explain the high prevalence of cribra orbitalia and porotic hyperostosis (82). In Italy, the prevalence of cribra orbitalia in Roman skeletal remains from the regions of Ravenna and Rimini dating from the 1st to the 4th century AD suggests that the observed lesions were caused by chronic acquired anemia, possibly caused by malaria. The authors have indeed argued that this area was surrounded by marshes during the Roman period (83). An osteological study conducted in Sardinia for the so-called “malaria signature” on 283 skeletons dating from 4,700 BC to 1582 AD, supported the possible introduction of malaria by Carthaginians during the 6th to 3rd century BC (84). Porotic hyperostosis is the consequence of thalassemia resulting in an overgrowth of the spongy marrow space of the skull. Accordingly, Angel supported the hypothesis that hemolytic anemia occurred under the pressure of *P. falciparum* (85, 86). In the regions of Cyprus, Greece, and Natalia, researchers identified the presence of Porotic hyperostosis on ten skeletons dating from 6,500 to 2,000 BC; some infantile long bones presented an internal shell of bone attached to the cortex, indicating that the red marrow hypertrophy blocked normal bone remodeling, resulting in damage caused by thalassemia (85). However, several researchers refute the hypothesis that cribra orbitalia can be caused by malaria infection. According to work conducted by Cole and Waldron in 2019, there was no direct and specific relationship in the blood dyscrasias causing anemia for which malaria agents are possibly responsible. Cribra orbitalia can then be considered as a non-specific paleopathological sign of malaria (87).

Paleogenetic approach has helped anthropologists to understand the evolution of malaria in Europe, malaria prevalence, genetic selection and ecological changes (88). Beta-thalassemia is a hemoglobinopathy with a specific mononucleotide mutation in the B-globin chain, which could confer protection against malaria following natural selection (89). The factors influencing thalassemia are diverse but the most important is undoubtedly resistance to malaria, indeed the distribution of malaria and the high prevalence of β-thalassemia are closely linked (90). The incidence rate of β-thalassemia in Sardinia is one of the highest in Europe with a majority of a cod39 mutation. Studying b-globin mutations by PCR analysis in 19 individuals dating from the Roman and Punic periods, identified the cod39 mutation in one individual, which was interpreted as clue for malaria endemicity during the Roman period in Sardinia (91). Also, complementary receptor 1 (CR1) involved in the interaction between Plasmodium and cells, is taken as an index of malaria susceptibility or protection (92). Studying CR1 single nucleotide polymorphisms (SNPs) in the Sardinian population found the prevalence of the dominant Sardinian haplotype was more closely related to that in India, than to other European ethnic groups: this observation evoked a positive selection supporting a malaria endemicity in the past (93). Another study found a high incidence of HLA-B35 in the Sardinian population, indicating that *P. falciparum* had probably exerted a selective and constant pressure for millennia on the Sardinian villages of low altitude (94).

### Malaria Eradication Strategies in Historical Europe

Since antiquity, malaria In Europe has been associated with swamps and stale air. Consequently, many strategies have been implemented to try to clean up these high-risk areas. French authorities opted for a policy of draining marshes as early as 1599 (Edict of King Henry IV for the draining of marshes) and a grand campaign started at that time everywhere in France (95). The King called upon the Dutch engineer Hunfroy Bradleij, who was experimenting in draining marshes. In 1643, during the reign of King Louis XIV (only 5 years old at the time, so it was his regents who decided), the King granted the owners of marshlands privileges for draining their land (95). In 1767, the extent of the marshes was estimated to be equivalent to a third of the kingdom. However, when the law of September 16th, 1807 related to draining marshes was implemented, a more realistic estimate indicated the extent of the marshes at 500,000 hectares. This decreased to 299,000 hectares in 1979 following various policies for draining marshes (96). A study conducted in Great Britain of factors influencing the eradication of malaria, based on analysis of data from 1840 to 1910, an increase in the cattle population was correlated with a 20% decrease in malaria, due to the decrease in wetlands following the extension of cattle breeding (97). The 2003 British study by Kuhn et al., based on statistical studies of the occurrence of fever epidemics between 1840 and 1910, simulated the presence of malaria during this period. The results indicated that mortality due to fevers would have been 19.5% higher if the density of cattle rearing and the extent of wetlands had remained unchanged between 1840 and 1910 (97). This study confirmed the importance of these parameters in playing a role in the eradication of malaria in Great Britain (97). In the southernmost regions of Europe, including Italy, the number of cases at the end of the 19th century amounted to 2 million, mostly in the Italian islands as well as in the central and southern regions, with a mortality rate of more than 15,000 deaths per year. It was not until the beginning of the 20th century that a major effort against malaria was undertaken with the passing of a law to control the *Anopheles* mosquito and the free distribution of quinine to the population (98). Italy was a pioneer and can be considered the testing ground for this approach to the struggle, which was later applied by the other European countries.

In 1928–1932, Benito Mussolini organized draining and cultivating the Pontine marshes at 60 km from Rome, considered as the main historical focus of malaria in central Italy. In 1943, faced with the advance of the Allies who landed in Sicily, Germans sabotaged the water infrastructure there in order to encourage the reappearance of malaria. During the second World War dichlorodiphenyltrichloroethane (DDT) was used extensively by the military to control malaria-carrying mosquitoes, and after the war a massive use of DDT was applied in Italy to stop the proliferation of malaria vectors (99).

*P. falciparum* and *P. vivax* were endemic in Corsica, a French Mediterranean mountainous island, just after the Second World War (100). The use of DTT associated with systematic oral administration of quinine lead to malaria eradication in 1953. Mosquito control measures were gradually abandoned. *P. vivax* outbreak re-emerged from 1964 to 1972 with the arrival of immigrants from North Africa (101). Prophylactic drug administration and insecticide spraying were re-established until the 1980s. The last case of autochthonous *P. vivax* was detected in 2006 (102). However, competent mosquitos for *P. falciparu*m malaria transmission are still present in Corsica and favorable climate may lead to the possibly re-emergence of malaria in Corsica (103).

Although the discovery of malaria parasites only occurred in the 19th century, when it was realized that malaria was not caused by bad air from the marshes but rather microorganisms in mosquitoes, this should not obscure the fact that malaria fevers existed in Europe and that they were very common and endemic in swampy areas (96).

### *Anopheles* and Malaria

The malaria parasite is transmitted to humans through the bite of female *Anopheles* mosquitoes, in which the parasite develops from the gametocyte to the sporozoite stage (104). After transmission to humans by mosquito bite the parasites develop in the liver. Later, they pass into the bloodstream, where they develop inside red blood cells, which are destroyed by the release of merozoites; it is during this stage that malaria is symptomatic (104). In 1818, J.W, Meigen described for the first time mosquitoes of the genus *Anopheles* (105). In 1897, Ronald Ross elucidated a missing step in the cycle of malaria parasites by discovering the role of mosquitoes in the transmission of malaria parasites (20). In Europe, several species of *Aedes, Culex*, and *Anopheles* mosquitoes, which are possible vectors of parasites of the genus *Plasmodium*, are present on the continent (106). A 2021 study looked at modeling of the zoogeographic history of possible malaria vectors in the Mediterranean region of Europe during the Quaternary periods. The results suggest the persistence of *P. vivax* and the *Anopheles* vector in central and southern Europe, supporting a permanent survival throughout the Quaternary period until the industrial revolution and subsequent eradication campaigns (107). In Europe, *An. maculipennis* has been considered as the major vector of malaria. However, a 2011 study warns about the ability of another urban species, such as *An. plumbeus*, which, according to the authors, may be a potential source of malaria transmission in urban areas (108). The spread of malaria in Europe was probably due to the spread of its vector, the *Anopheles* mosquito (13) (Box 3). This was confirmed by the role played by *A. gambiae* in Africa for the spread of malaria in that continent (109). In Italy during the Middle Ages, the spread of malaria probably followed the spread of the *A. sacharovi* mosquito in central and southern Italy (13). In the region of Lugnano, where the presence of malaria in the 5th century was demonstrated by molecular biology (13), the spread of *A. labranchiae* was considerable in the region during this period, playing the role of vector, later replaced by *A. atroparvus* in the beginning of the 20th century (13). Anthropological study disclosed traditional Sardinian culture traits limiting exposure to the malaria vector *Anopheles labranchiae*, recalled that Sardinia was probably the European territory most affected by malaria between the end of the 19th and the beginning of the 20th century (88, 110). *An. sacharovi* has disappeared in Corsica but *An. labranchiae*, which is competent in the transmission of *P. falciparum*, remains currently abundant in the island (103).

Box 3 **Malariotherapy.**

Chronic syphilitic meningoencephalitis causes a fatal progressive degeneration of the central nervous system known as general paralysis (111, 112). In the absence of any known effective treatment, Julius Wagner-Jauregg first used so-called malariotherapy in Vienna on June 14, 1917 (113). The goal of this therapeutic approach was to provoke a controlled fever to prevent complications from neurosyphilis. At the start of the trials, Wagner used erysipelas, which was a failure (114). He later tried different fever causative agents such as tuberculin, smallpox, and typhoid before opting for *P. vivax* inoculation, which was judged to be more controllable and curable by quinine administration (115). When Wagner-Jauregg attended a soldier with malaria returning from the Balkans, he saw it as a sign of fate, and after confirming that the soldier's blood was infected with *P. vivax*, he decided to inoculate it in three patients with general paralysis. These were the first trials of malariotherapy (116). For this work Wagner-Jauregg received the Nobel Prize in Medicine in 1927.

Pyrotherapy was reinforced by *Treponema pallidum* sensitivity tests to hyperthermia carried out in 1932 by Boak et al., demonstrating that *T. pallidum* was susceptible to heat above 37.5°C (117). In New York City between 1923 and 1927, Bunker and Kirby, treating general paralysis in a total of 156 patients, reported a complete remission rate of 33 vs. 26% mortality (114). In the absence of standards and controls, the results seemed promising at the time, giving hope for a cure of general paralysis (114). In 1941, after the introduction of penicillin as a treatment, malariotherapy stopped (115). Today, malariotherapy may be seen as horrible and unethical, but at the time, given the desperate cases caused by the neurological form of syphilis, resorting to this therapy seemed to be a godsend.

### Blood Groups and Malaria Susceptibility

Several studies have demonstrated a strong possible relationship between different blood groups of the different referenced grouping systems and malaria susceptibility. Epidemiological studies assessing the relationship between ABO blood grouping and malaria susceptibility indicate that individuals with group O are significantly protected against severe *P*. *falciparum* malaria (118–120).This is explained by the significant reduction in the cytoadherence of red blood cells, which is responsible for severe malaria syndromes, in individuals with group O compared to individuals with other groups (121, 122). Another grouping system most frequently used in the case of malaria is the Duffy system, and represents the presence or absence of Fy antigens (Fy a and Fy b) on the surface of red blood cells; these antigens are considered receptors for the parasites *P. vivax* and *P*. *knowlesi*. The absence of the Duffy antigen (Fy a - b- phenotype), which is dominant in Africa, confers therefore some resistance against *P*. *vivax* and *P. knowlesi* (123). Finally, another blood grouping system of interest is the Gerbich system, where studies have shown that the rate of individuals positive for *P*. *falciparum* and/or *P. vivax* is more frequently found in Gerbich “Ge-negative” individuals, suggesting that the negativity of the Ge antigen protects against malaria infection and confers some resistance (124). This may be reflected in the fact that the absence of c-glycophorins and thus of proteins 4.1 and P55, which leads to alterations in the skeleton of the erythrocyte membrane, limits the invasion of malaria merozoites (125).

## Conclusion

Current paleomicrobiological diagnostic methods are robust enough to support and confirm symptoms suggesting the occurrence of pandemic malaria during different historical periods, as described by ancient texts, using direct detection of infectious agents in ancient specimens; and to enter the differential diagnosis of malaria with mimicking infectious such as among others, pyogenic infections, tuberculosis, schistosomiasis, leptospirosis or borreliosis (126). In Europe, studies based on molecular techniques of malaria detection are still few and mainly concentrated in Italy. However, these studies support the ability to detect ancient malaria DNA in the old World. The development of molecular techniques is necessary to improve parasite detection of *Plasmodium* genus from human remains. The development of direct detection of ancient malaria would be an excellent way to study the origin, evolution, and frequency of malaria in Europe over the centuries. In this objective, several archaeological sites seem interesting for the study of malaria occurrence based on ancient texts and climatic and environmental parameters, like wet and formerly swampy areas such as Versailles, Corsica, and Sologne in France.

In view of current and future climate changes and variability in the European continent, understanding the evolution of malaria, its *Anopheles* vectors and the climatic parameters that were conducive to its expansion, is particularly important in understanding host-pathogen interactions, modes of transmission, and frequency of spread. This would provide a good basis, given the possibility of malaria resurgence in a currently malaria-free Europe, for developing a strategy to avoid its resurgence. For instance, competent mosquitos for *P. falciparu*m malaria transmission (*An. labranchiae*) are still present in Corsica and favorable climate may lead to the possibly re-emergence of malaria in Corsica (103).

## Author Contributions

MB, BP, MD, and RB wrote the manuscript. MB and RB designed the figures. All authors contributed to the article and approved the submitted version.

## Conflict of Interest

The authors declare that the research was conducted in the absence of any commercial or financial relationships that could be construed as a potential conflict of interest.
